# Transcriptome profiling of pyrethroid resistant and susceptible mosquitoes in the malaria vector, *Anopheles sinensis*

**DOI:** 10.1186/1471-2164-15-448

**Published:** 2014-06-09

**Authors:** Guoding Zhu, Daibin Zhong, Jun Cao, Huayun Zhou, Julin Li, Yaobao Liu, Liang Bai, Sui Xu, Mei-Hui Wang, Guofa Zhou, Xuelian Chang, Qi Gao, Guiyun Yan

**Affiliations:** Department of Parasitology, Medical College of Soochow University, Suzhou, 215123 PR China; Jiangsu Institute of Parasitic Diseases, Key Laboratory of Parasitic Disease Control and Prevention (Ministry of Health), Jiangsu Provincial Key Laboratory of Parasite Molecular Biology, Wuxi, Jiangsu Province 214064 PR China; Program in Public Health, College of Health Sciences, University of California at Irvine, Irvine, CA 92697 USA

**Keywords:** Transcriptome, Expressed sequence tag, Pyrethroid resistance, Gene expression, *Anopheles sinensis*

## Abstract

**Background:**

*Anopheles sinensis* is a major malaria vector in China and other Southeast Asian countries, and it is becoming increasingly resistant to the insecticides used for agriculture, net impregnation, and indoor residual spray. Very limited genomic information on this species is available, which has hindered the development of new tools for resistance surveillance and vector control. We used the 454 GS FLX system and generated expressed sequence tag (EST) databases of various life stages of *An. sinensis*, and we determined the transcriptional differences between deltamethrin resistant and susceptible mosquitoes.

**Results:**

The 454 GS FLX transcriptome sequencing yielded a total of 624,559 reads (average length of 290 bp) with the pooled *An. sinensis* mosquitoes across various development stages*.* The *de novo* assembly generated 33,411 contigs with average length of 493 bp. A total of 8,057 ESTs were generated with Gene Ontology (GO) and Kyoto Encyclopedia of Genes and Genomes (KEGG) annotation. A total of 2,131 ESTs were differentially expressed between deltamethrin resistant and susceptible mosquitoes collected from the same field site in Jiangsu, China. Among these differentially expressed ESTs, a total of 294 pathways were mapped to the KEGG database, with the predominant ESTs belonging to metabolic pathways. Furthermore, a total of 2,408 microsatellites and 15,496 single nucleotide polymorphisms (SNPs) were identified.

**Conclusions:**

The annotated EST and transcriptome databases provide a valuable genomic resource for further genetic studies of this important malaria vector species. The differentially expressed ESTs associated with insecticide resistance identified in this study lay an important foundation for further functional analysis. The identified microsatellite and SNP markers will provide useful tools for future population genetic and comparative genomic analyses of malaria vectors.

**Electronic supplementary material:**

The online version of this article (doi:10.1186/1471-2164-15-448) contains supplementary material, which is available to authorized users.

## Background

The malaria vector mosquito, *Anopheles sinensis* (Diptera: Culicidae) is widely distributed in China and other Southeast Asian countries [1]. Due to its relatively high zoophilic and exophilic behaviors, it was treated as a secondary vector for many years in the 20^th^ century. However, the outbreak of *Plasmodium vivax* malaria in central China in which *An. sinensis* was the only vector leads us to reassess its role for malaria transmission [2, 3]. Consequently, increased attention has been shifted to *An. sinensis* due to its wide distribution, high abundance and modest susceptibility to malaria parasites. However, little genetic information is available for this species, which has significantly limited the further research and development of new vector control tools.

Vector control is a critical component of all malaria control strategies [4, 5]. Currently, it relies primarily on two interventions: long-lasting insecticide nets (LLIN) and indoor residual spraying (IRS) [6]. Due to their low toxicity and high efficacy, pyrethroids are the only insecticide approved by the World Health Organization (WHO) for bed net impregnation, and they are used often for IRS [7]. The significant increase in insecticide-based malaria vector control in the past decade has resulted in rapid spread of resistance among malaria vectors, which has placed current global efforts in malaria control and elimination at risk [8–10]. In 2012, WHO launched a Global Plan for Insecticide Resistance Management in malaria vectors (GPIRM), which advocates the collection of baseline information on insecticide resistance at the global scale and the development of novel methods to further understand the molecular mechanism of insecticide resistance and to enhance resistance surveillance [11]. In China, deltamethrin and permethrin were the major pyrethroid insecticides for vector control, and pyrethroid resistance was reported in limited number of *An. sinensis* populations in the 2000s [12]. However, deltamethrin resistance has been found in *An. sinensis* from southern and central China where malaria is endemic or epidemic [13–16]. Resistance to various classes of insecticides was also reported in *An. sinensis* in South Korea [17–19].

Two types of pyrethroid insecticide resistance mechanisms have been recognized, including alteration of the target sites in the *para*-type sodium channel that leads to knockdown resistance (*kdr*) and up-regulation of insecticide detoxification enzymes such as P450 monooxygenases, glutathione S-transferases and carboxylesterases [20]. For *An. sinensis*, high *kdr* mutation frequencies have been reported in central China (Hunan, Hubei, Henan and Anhui provinces) whereas Yunan populations in southwestern China completely lacked *kdr* mutations [13, 16]. Overall, metabolic detoxification enzyme plays an important role in pyrethroid resistance [13, 16]. Recent studies using the microarray hybridization method suggest that cuticle-related genes may also be associated with resistance [21]. The recent development of the RNA-seq method enables us to determine gene expression patterns while providing *de novo* sequencing, assembly and annotation of expressed genes [22]. The RNA-seq method is particularly suitable to species like *An. sinensis* which do not have a complete genome sequence, as it can help with developing expressed sequence tag (EST) databases. Various sequencing platforms (e.g., Roche 454, Solexa/Illumina, ABI SOLiD, etc.) are available, each having its own advantages [22, 23]. For example, Roche 454 sequencing technology provides longer sequence reads, and thus it is useful for genome sequence assembly and developing EST databases (e.g., *An. funestus*[24], *Aedes aegypti*[25], *Bactrocera dorsalis*[26], *Melitaea cinxia*[27], *Zygaena filipendulae*[28], *Chrysomela tremulae*[29], *Aphis glycines*[30], *Cimex lectularius*[31], *Manduca sexta*[32, 33], *Laodelphax striatellus*[34], *Stomoxys calcitrans*[35], *Dermacentor variabilis*[35], *Erynnis propertius* and *Papilio zelicaon*[36], *Lygus hesperus*[37] and *Agrilus planipennis*[38]).

The objectives of this study were to enrich the genetic information of *An. sinensis,* an important malaria vector for genetic and comparative genomic studies, and to enhance understanding of insecticide resistance mechanism. We constructed a reference transcriptome of *An. sinensis* using the 454 GS FLX system with mosquito samples from different developmental stages, generated an EST database, and examined the transcriptome profiles between deltamethrin resistant and susceptible mosquitoes.

## Methods

This study used *An. sinensis* mosquitoes collected from the field and laboratory susceptible colony. Field-collected *An. sinensis* mosquitoes were phenotyped for susceptibility to deltamethrin resistance and used for transcriptome profile determination in the resistant and susceptible populations. The laboratory susceptible colony was primarily used to generate reference expressed sequence tag (EST) databases.

### Field mosquito sample collection and insecticide susceptibility bioassay

**Figure 1:**
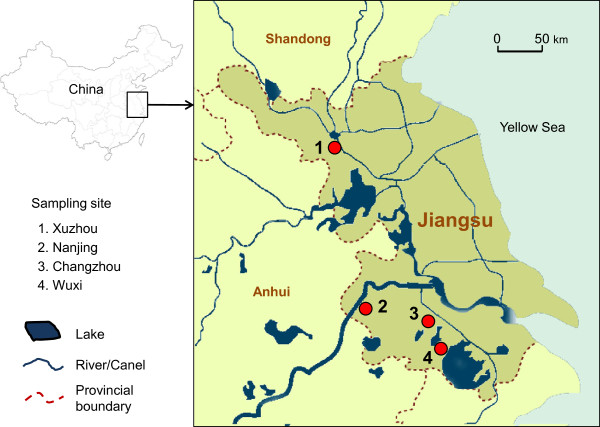
**Map of** ***Anopheles sinensis*** **mosquito sampling sites in Jiangsu Province, China.** Red filled circles represent the sampling sites of the four major cities: Xuzhou city, Nanjing city, Changzhou city, and Wuxi city.

Anopheline mosquito larvae and pupae were collected from rice fields in four locations: Xuzhou, Nanjing, Changzhou and Wuxi of Jiangsu Province, China, between July and September, 2011 (Figure 1). A total of 30–50 breeding sites were used per site. The study area has been experiencing sporadic *P. vivax* malaria outbreaks. Since the 1980s, deltamethrin impregnated nets (ITNs) and indoor residual spraying (IRS) have been widely used to control mosquitoes. Rice is the major agricultural crop in these study sites. Due to severe insect pest damage to the rice crop, insecticide use for rice pest control has been very intensive, with several rounds of sprays in each growing season. Pyrethroids are commonly used for agricultural pest control in study areas, but other insecticides such as organic phosphates and carbamates are also being used.

The field-collected mosquito larvae and pupae were reared to adulthood in the insectary of the Key Laboratory on Technology for Parasitic Disease Control and Prevention, Ministry of Health, Jiangsu Institute of Parasitic Diseases (JIPD) in Wuxi, Jiangsu Province, China. The average room temperature and relative humidity were 27 ± 1°C and 70 ± 10%, respectively. After the mosquitoes were identified to species morphologically, female adults of *An. sinensis* 3 days post emergence were tested for susceptibility to deltamethrin using the standard WHO tube bioassay with 0.05% deltamethrin test papers [39]. Between 100 and 200 mosquitoes per location were tested with 20 mosquitoes per tube, providing 5–10 biological replicates. The knockdown time was recorded for all tested mosquitoes at 10-minute intervals during the 60 minute exposure time. All mosquitoes alive or dead 24 hours after the recovery period were preserved in 100% ethanol or RNAlater (Qiagen) for subsequent molecular analyses. WHO [39] criteria were used to classify resistance status of the tested mosquito population (resistant if mortality is <90%, probable resistant if mortality is 90–98%, and susceptible if mortality is >98%) [39].

### Molecular identification of mosquito species and *kdr* genotyping

Genomic DNA was extracted from single mosquito legs for molecular identification and *kdr* genotyping of *An. sinensis.* A total of 514 field-collected mosquitoes were identified to species using the ribosomal DNA internal transcribed spacer 2 (rDNA-ITS2)-based method [40], and *kdr* genotyping of the field-collected *An. sinensis* mosquitoes followed our previous established allele-specific PCR (AS-PCR) method [16].

### Laboratory colony mosquito sample preparation for reference construction

To obtain reference EST information for *An. sinensis,* we used mosquitoes from all development stages (eggs, first to fourth instar larvae, pupae, and female adults) of an *An. sinensis* laboratory colony maintained at JIPD. A total of 100 eggs, 20 larvae, 20 pupae, and 20 female adult mosquitoes of the *An. sinensis* laboratory colony were used in the study. This laboratory colony was never exposed to any insecticide and was highly sensitive to deltamethrin.

### RNA-seq library preparation and 454 sequencing

To maximize transcripts coverage, we constructed 4 RNA libraries from *An. sinensis* mosquitoes of different development stages of JIPD laboratory susceptible colony, and from field collected, deltamethrin-resistant and susceptible mosquitoes. The first library was pooled RNA samples from 100 eggs, 20 larvae and 20 pupae of the *An. sinensis* laboratory colony. Thus this library targeted mosquito immature stages. The second library was RNA samples from 20 female adults, four to five days post emergence, of the *An. sinensis* laboratory colony. The third library was made from 20 female adult mosquitoes reared from field-caught larvae, four to five days post emergence, phenotypically identified as deltamethrin resistant by using the standard WHO tube assay. The fourth library was made from 20 female adult mosquitoes reared from field-caught larvae, four to five days post emergence, phenotypically identified as deltamethrin susceptible. Mosquitoes for the third and fourth libraries were from the same field sites in Jiangsu Province. Data from all four libraries were used to develop the EST database. Comparison of RNA-seq data between the third and fourth libraries would reveal transcriptome differences associated with deltamethrin resistance.

Field-collected deltamethrin-resistant individuals were confirmed by the WHO insecticide-susceptibility bioassay. The mosquitoes that survived after the 24 hr recovery period were classified as deltamethrin resistant, and those knocked down early during the bioassay were classified as deltamethrin susceptible [41]. This classification is reasonable because the discriminant dosage used in the bioassay (0.05% deltamethrin) kills 99.9% of susceptible mosquitoes [39]. We used deltamethrin resistant and susceptible mosquitoes from the same area under the same growing conditions to reduce the confounding effects of geographic origin of the specimens and larval growing conditions.

Total RNA of *An. sinensis* was extracted from the deltamethrin resistant and susceptible mosquitoes using the PureLink RNA Mini Kit (Life Technologies). Similar procedures were used to extract the total RNA of various stages of the laboratory colony *An. sinensis* mosquitoes used in the EST discovery. Total RNA quantity was assessed using the NanoDrop spectrophotometer (NanoDrop Technologies), and RNA integrity was examined by running on 1.2% agarose gel with the treatment of RNase Zap (Sigma-Aldrich) followed by the Agilent 2100 Bioanalyzer.

The full-length cDNA for each library was synthesized and amplified using the Mint-2 cDNA synthesis kit (Evrogen, Moscow, Russia). Briefly, the first strand of the cDNA was synthesized using a mixture of 0.5 μg total RNA, 1 μl PlugOligo-3 M adapter at 15 μM (5′-AAGCAGTGGTATCAACGCAGAGTGGCCATTACGGCCGGGGG-3′), and 1 μl 3′-end CDS-Gsu adapter at 10 μM (5′-AAGCAGTGGTATCAACGCAGAGTACTGGAG(T)20VN-3′). Because the first strand cDNA synthesis started from a 3′-end CDS adapter that contained an oligo (dT) 20 sequence which anneals to poly(A) stretches of mRNA, this procedure selected mRNA and helped filtering out rRNA. The mixture was incubated at 70°C for 2 min, and then the incubation temperature was decreased to 42°C. After incubation, each mixture was added to 5 μl reverse transcription solution (2 μl 5X first strand buffer, 1 μl DTT at 20 mM, 1 μl dNTPs at 10 mM each, and 1 μl mint reverse transcriptase), incubated at 42°C for 30 min, and then incubated with IP-solution (solution for incorporation of the PlugOligo sequence) for 1.5 hr at 42°C. Next, the double-stranded cDNA was synthesized in a 50 μl reaction volume containing 40 μl sterile RNase-free water, 5 μl 10X Encyclo buffer, 1 μl dNTP mix (10 mM each), 2 μl PCR primer at 10 μM (5′-AAGCAGTGGTATCAACGCAGAGT-3′), 1 μl first-strand cDNA, and 1 μl 50X Encyclo polymerase mix. The amplification was performed with the following conditions: one cycle at 95°C for 1 min, followed by 18 cycles at 95°C for 15 sec, 66°C for 20 sec, and 72°C for 3 min.

Our first library was used for EST discovery; therefore, this library was normalized using the Trimmer-2 cDNA normalization kit (Evrogen) to minimize over-presentation of some abundant transcripts. The CDS-Gsu adapter was used for normalization prior to the cDNA library construction. The other three libraries were not normalized because our objective was to determine the transcriptome profiles in field-collected deltamethrin resistant or susceptible mosquitoes and in laboratory-reared susceptible mosquitoes. In this case, the CDS-Gsu adapter was replaced by the CDS-4 M adapter (5′-AAGCAGTGGTATCAACGCAGAGTGGCC GAGGCGGCC(T)4G(T)6C(T)13VN-3′). Each RNA-seq library was sequenced using the 454 GS FLX by the Center for Integrated Biosystems, Utah State University, USA.

### RNA-seq data analyses

#### *De novo* transcriptome assembly and functional annotation

We first trimmed the adapter sequences from the original reads of the 454 GS FLX and removed the reads with low quality and short sequences (<50 bp). *De novo* assembly was conducted for the individual libraries and for the four libraries pooled to maximize the coverage and to provide a more comprehensive reference transcript. The *de novo* assembly was generated using the CLC Genomics Workbench version 6.0 (CLC Bio) with default parameters (similarity = 0.8, length fraction = 0.5, insertion/deletion cost = 3, and mismatch cost = 3) [42]. The contigs of >200 bp assembled from pooled libraries were BLASTXed against the nr (non-redundant) protein database of the National Center for Biotechnology Information (NCBI), and the taxonomic distribution of hits matching the nr protein database was determined. BLAST output was post-processed and lists of hits with match locations, e-values and bit scores were generated. A cutoff e-value of 1e-5 was used. Gene Ontology (GO) annotations were conducted by using the Blast2GO program [43], and the WEGO software was used to display GO functional classification [44].

### Homology-based EST filtering

To determine *An. sinensis* gene homology to other previously sequenced mosquito species, a BLASTN search against *An. gambiae*, *Aedes aegypti* and *Culex quinquefasciatus* transcript-protein sequences in VectorBase database (http://www.vectorbase.org) was conducted. BLASTN results were parsed so that contigs with an E-value <0.001 in bi-directional best hit were retained for each query. Each homolog of the gene pair was then searched with TBLASTX against the three genomes. ESTs matching with an E-value <1 × 10^−5^ were considered putative homologs. Identical top BLAST hits suggested putative orthologs, whereas non-identical top BLAST hits suggested the possible presence of paralogs, which were excluded from the analysis. The matched subsequence of the query contig that had the longest aligned region among all queries was used as the candidate transcript. Finally, identical candidate ESTs were collapsed into unique EST sequences.

### Identification of differentially expressed genes (DEGs)

To compare the transcriptomes from field mosquitoes and library colony, and the resistant and susceptible mosquitoes, the trimmed reads from the second library (library colony), the third (deltamethrin- resistant) and fourth library (deltamethrin-susceptible) were mapped and aligned to the final EST set using the CLC Bio software. The following mapping parameters were used: mismatch cost = 2, insertion cost = 3, deletion cost = 3, length fraction = 0.5, similarity fraction = 0.8. Gene expression was quantified by RPKM (reads per kilobase per million mapped reads) to minimize the influence of variation in gene length and total number of reads [42]. RPKM values were log2 (RPKM + 0.0001) transformed, and average linkage clustering was used. Genes were classified as differentially expressed if they exhibited two folds or greater changes between the two samples, and statistical significance at P < 0.01 based on the Audic-Claverie method [45–47] and the false discovery rate (FDR) < 0.01 [48].

### Microsatellite and SNP discovery

The contigs assembled from the four library sequence reads were searched for microsatellite repeats using the MsatCommander software [49]. A microsatellite was identified if the contig contained a motif that was repeated at least six times for dinucleotides and at least four times for tri-, tetra-, penta-, and hexanucleotides. Candidate SNPs were identified from assembled reads using the CLC Bio software with quality-based variant detection [42]. Parameters were as follows: neighborhood radius = 5, maximum gap and mismatch count = 2, minimum neighborhood quality = 15, minimum central quality = 20, minimum coverage = 10, and minimum variant frequency (%) = 35.0. Putative open reading frames (ORFs) of assembled transcripts with a minimum size of 100 bp were predicted using the GETORF program in the EMBOSS package [50]. The longest putative ORF from any particular sequences were selected. When two similarly long (within 90% of each other) ORFs were found located in opposite strands of the contigs, we followed the method of Pellino [51] by comparing the polarity of the ORFs with the strand orientation from the Blastx analysis. A custom Perl script was used to test whether these SNPs resulted in an amino acid change in the predicted ORF.

### Experimental validation of RNA-seq data

Seven differentially expressed transcripts between the deltamethrin resistant and susceptible mosquitoes identified from RNA-seq were selected for independent validation of gene expression using quantitative real-time PCR (qRT-PCR) [52]. We chose these genes because of their important functions in insecticide resistance and mosquito immunity. Twenty *An. sinensis* female adults reared from field-collected larvae that were phenotyped as deltamethrin resistant or susceptible by the WHO tube bioassay were used. Quantitative real-time PCR reactions were performed in triplicate with LightCycler® 480 SYBR Green I Master. Fold changes in gene expression between resistant and susceptible mosquitoes were derived by the comparative CT method using the 18S ribosomal protein gene as an internal control [52]. Gel electrophoresis of the PCR products was also conducted to verify that a single gene-specific product was produced.

### Data deposition

The Roche 454 reads of *An. sinensis* obtained in this study were submitted to the NCBI Sequence Read Archive under the BioSample accession number of SAMN01892512 - SAMN01892515, and the Transcriptome Shotgun Assembly contigs (length ≥ 200 bp) were deposited to DDBJ/EMBL/GenBank under the accession number GAFE01000001- GAFE01028133 (Bioproject 186896).

### Ethics statement

No specific permissions were required for the described field studies. For mosquito collection in rice paddies, oral consent was obtained from field owners in each location. These locations were not protected land, and the field studies did not involve endangered or protected species.

## Results

### Susceptibility to deltamethrin and *kdr* genotyping

A total of 514 female mosquitoes reared from field-collected larvae were bioassayed for resistance to deltamethrin. We found a high level of resistance in the four mosquito populations because the mortality rate was all <10% whereas the laboratory susceptible strain showed 100% mortality (Additional file 1). PCR analysis with 514 specimens confirmed that all mosquito specimens examined in the bioassay were all *An. sinensis*. Three types of *kdr* mutations at codon position 1014 of the *para*-type sodium channel gene were detected: two mutations (from TTG to TTT and from TTG to TTC) that cause non-synonymous changes from leucine to phenylalanine (L1014F), and one mutation from TTG to TGT that causes leucine to cysteine substitution (L1014C). The *kdr* mutation frequency was high (88–100%) among the four tested populations and the predominant *kdr* mutation was L1014F. No *kdr* mutation was detected in the susceptible laboratory strain (Additional file 1).

### Assembly of contigs

**Figure 2:**
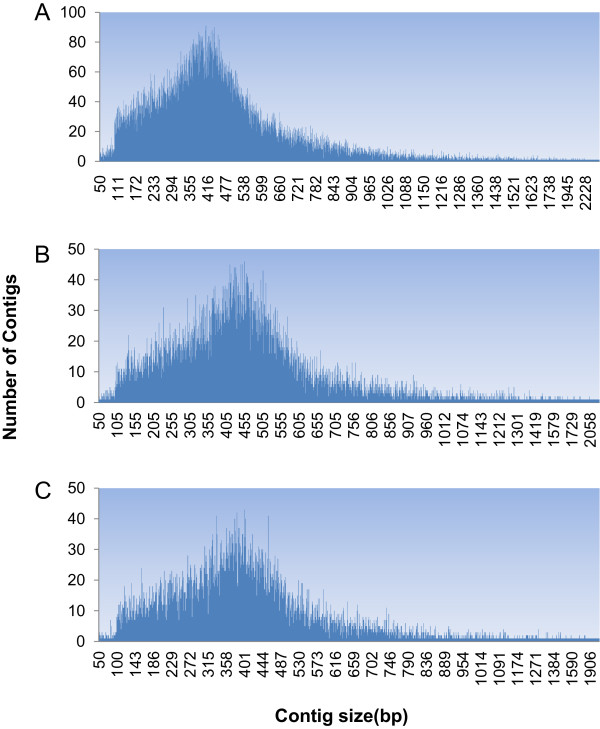
**Contig size distributions of** ***Anopheles sinensis.*** **A**: laboratory colony and field population (all four groups); **B**: Deltamethrin resistant female adults collected from the field; **C**: Deltamethrin susceptible female adults collected from the field.

The Roche 454 GS FLX platform produced a total of 624,559 reads from the 4 libraries. The average raw read length was 290.8 bp. After removal of duplicated, ambiguous and short (<50 bp) sequences, a total of 394,254 reads were used for EST contig assembly (Table 1). The average percentage of reads mapped to contig sets was 71.9%, producing a total of 33,411 EST contigs with an average length of 493 bp. Among them, 31,772 contigs were longer than 50 bp, 28,133 contigs were longer than 200 bp (Figure 2, GenBank accession: GAFE01000001- GAFE01028133).

**Table 1:** **Summary of reads and assembly from 454 GS FLX sequencing for** ***Anopheles sinensis***

Library	Raw reads	Average length (bp)	Reads used in assembly*	% reads mapped to contig set	No. contigs	N50 contigs (bp)	Average contig length (bp)	% GC contigs
Immature stage, colony	164,503	341.1	122,661	75.45	17,398	491	447	45.3
Female adults, colony	153,568	299.0	106,268	71.1	7,415	515	453	44.5
Deltamethrin resistant, field	171,679	363.3	124,723	73.0	13,897	524	481	45.5
Deltamethrin susceptible, field	134,809	304.6	91,193	68.3	10,597	464	427	45.2
Total	624,559	290.8	394,254	71.9	33,411	547	493	45.1

*Reads used in assembly is less than the total number of raw reads due to removal of duplicated, ambiguous and short (<50 bp) sequences.

### Homology-based EST filtering and EST function annotation

**Figure 3:**
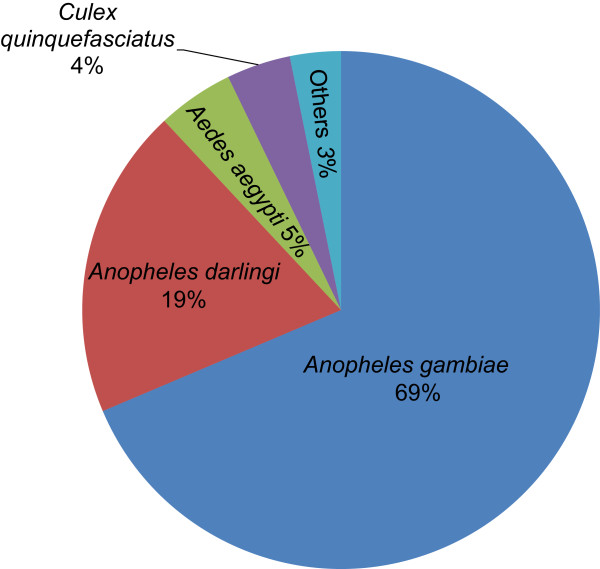
**Taxonomic distribution of the most hit species by BLASTX against National Center for Biotechnology Information (NCBI) nucleotide database.** Pie chart shows the percentage of contigs with top hits in various species with E-value cutoff at 1e-5.

To determine the taxonomy distribution of the assembled contigs, all 31,772 EST contigs longer than 50 bp were BLASTXed against the NCBI nr (non-redundant protein sequences) database using the expected value of 1e-5. We found that 10,504 unique contigs were matched to the nr database, with the most hits matching to *An. gambiae*, the most important malaria vector in Africa, followed by other mosquito species and insects (Figure 3, Additional file 2).

When we searched 31,772 assembled contigs against transcript databases of three mosquito species (*An. gambiae*, *Ae. aegypti* and *Cx. quinquefasciatus*) with the searching match criteria described in the method by BLASTN, 8,057 ESTs were identified. Among these, 5,545 were from laboratory adults and field-collected mosquitoes (Table 2). These ESTs showed >94% matched hits with AGAP (*An. gambiae*), consistent with BLAST-nr search results in which the dominant match was with *An. gambiae*.

**Table 2:** **Number of expressed sequence tags (ESTs) matching to reference mosquito transcript**

	Number of matches to			
Reference organism	Final EST set	Female adult, colony	Deltamethrin resistance, field	Deltamethrin susceptible, field
*Anopheles gambiae*	6,781	1,496	2,000	1,743
*Aedes aegypti*	471	40	53	47
*Culex quinquefasciatus*	805	52	58	56
Total number of matches	8,057	1,588	2,111	1,846

### Gene Ontology (GO) classification and Kyoto Encyclopedia of Genes and Genomes (KEGG) analysis

**Figure 4:**
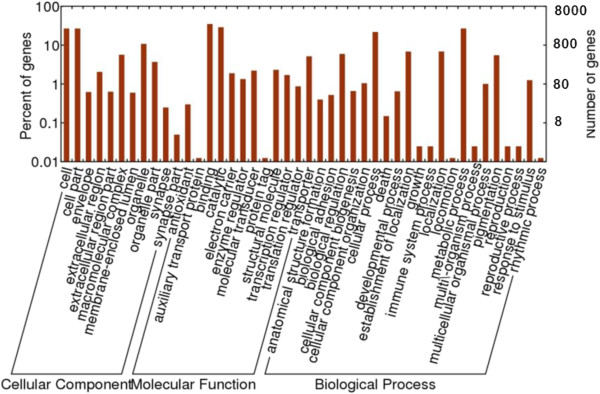
**Histogram of** ***Anopheles sinensis*** **expressed sequence tag (EST) Gene Ontology (GO) classification.** Three main ontologies of GO (biological process, cellular component and molecular function) are shown in the x-axis. The left y-axis indicates the percentage of total genes and the right y-axis is the number of genes in each category.

The 8,057 ESTs were assigned to GO accession numbers (Additional file 3), and they were classified into 44 function categories under three major domains (biological process, cellular component, and molecular function; Figure 4). To investigate the biological function, all 8,057 ESTs were mapped to the reference pathways in the KEGG. A total of 2,241 ESTs obtained KEGG annotation, and 1,957 ESTs were assigned to 289 KEGG pathways. A large number of ESTs were restricted to single pathways, such as metabolic pathway (390 ESTs), biosynthesis of secondary metabolites (122 ESTs), and microbial metabolism in diverse environment (74 ESTs).

### Differentially expressed genes associated with deltamethrin resistance and pathway analyses

**Figure 5:**
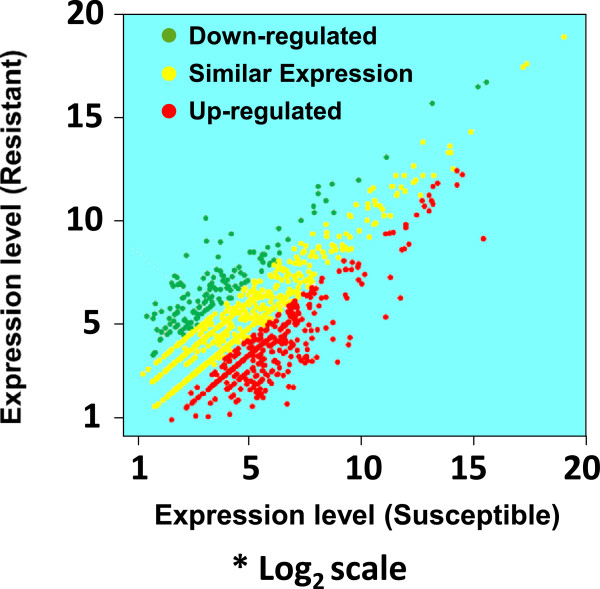
**Scatter plots of differentially expressed genes between deltamethrin resistant and susceptible** ***Anopheles sinensis*** **mosquitoes.** The y-axis indicates the expression level of resistant mosquitoes and the x-axis is the expression level of susceptible mosquitoes. Transcription is expressed as log2-transformed reads per kilobase per million reads (RPKM + 0.0001).

**Figure 6:**
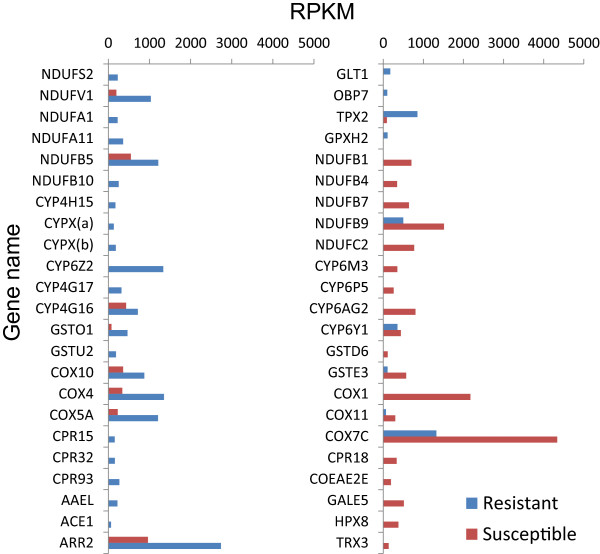
**Distribution of expression values in genes involved in metabolic detoxification and insecticide penetration in deltamethrin resistant and susceptible** ***Anopheles sinensis*** **mosquitoes.** RPKM refers to the reads per kilobase per million mapped reads.

Comparisons between field deltamethrin-resistant adult mosquitoes and adult from the susceptible laboratory colony identified a total of 2,088 (25.9%) transcripts that were expressed at 2 folds or greater changes with P-value < 0.01 and FDR < 0.01) (Additional file 4). Among them, 1,197 (14.8%) were up-regulated and 891 (11.1%) down-regulated in the field resistant mosquitoes. Comparison of RNA-seq data with samples between field resistant and field susceptible mosquitoes, we found a total of 2,131 differentially expressed transcripts, 1,079 (13.4%) up-regulated and 1,052 (13.1%) down-regulated in resistant mosquitoes (Figure 5, Additional file 4). Among them, 930 (11.5%) transcripts were expressed only in resistant mosquitoes, while 665 (8.3%) transcripts expressed only in susceptible mosquitoes. Among these significantly differentially expressed genes, 1,286 showed significant matches to the KEGG database, and 916 genes were mapped to 294 pathways. The remaining 370 genes did not have the related genes annotation and KO number when searching with KEGG Mapper (http://www.genome.jp/kegg/tool/map_pathway2.html). The most commonly identified pathway was “metabolic pathway,” which was previously found to be involved in insecticide metabolism [53–56]. Among the different transcripts, the majority were in the families of cytochrome P450 monooxygenases (21.7%), NADH dehydrogenase (21.7%), and glutathione S-transferase (8.7%) (Figure 6). The transcripts with the largest increases in expression were also in the metabolic pathway (Additional file 5), especially the genes coding for P450 monooxygenases, GSTs, and the NADH dehydrogenase, a cofactor of P450 monooxygenases. Mitochondrial cytochrome C oxidase and the cuticular proteins exhibited significantly differential expression between deltamethrin resistant and susceptible mosquitoes.

### Verification of RNA-seq data by quantitative RT-PCR

The qRT-PCR reactions were carried out with 6 transcripts identified by RNA-seq as differentially expressed between deltamethrin resistant and susceptible mosquitoes. The gene name, function and primer sequences used for qRT-PCR amplification for RNA-seq validation are shown in Additional file 6. When expression data from RNA-seq were compared to qRT-PCR, the direction of fold changes detected by these two methods were consistent for all 6 tested transcripts, although the RNA-seq method generally identified higher numbers of fold changes (Additional file 7).

### Microsatellite discovery

**Figure 7:**
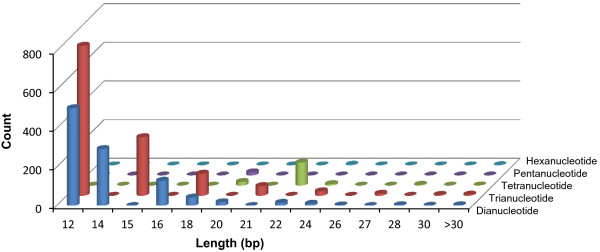
**Distribution of microsatellites in** ***Anopheles sinensis*** **expressed sequence tags (ESTs).** The y-axis indicates the number of sequences and the x-axis is the length of repeats.

The assembled 31,772 contigs were used for *An. sinensis* microsatellite identification. Overall, a total of 2,408 di-, tri-, tetra-, penta-, and hexanucleotide microsatellites from 2,225 contigs were identified on the conditions that these microsatellites had a minimum 6, 4, 4, 4, and 4 repeat units and each microsatellite was found in at least 5 contigs. The distributions of microsatellites in these assembled contigs are shown in Figure 7. The dinucleotide repeats were the most abundant (51.7%), followed by trinucleotide and tetranucleotide repeats (41.4%). The AC and GT dinucleotide repeats were the most abundant (29.7%) classes of repeats, while the CG repeats were the least abundant. Among trinucleotide microsatellites, AGC repeats were the most abundant, while CGG repeats were the least abundant.

### SNP discovery

The present study used pooled field mosquito individuals for 454 sequencing, and thus it offered a good opportunity to identify SNPs in the transcribed sequences of *An. sinensis.* The criteria for SNP identification were at least two occurrences of the minority allele at sites covered by at least ten sequences. We found a total of 15,496 SNPs in the pooled transcriptome, providing an estimation of approximately 6 SNPs per 1000 bp with an average coverage of 28. The majority of the SNPs (60%) were transitions (C↔T and A↔G). The percentage of transversions ranged from 7.2% (G↔C) to 14.0% (A↔T). To distinguish synonymous and non-synonymous SNPs, the GETORF program was used to identify open reading frames. A total of 34,918 open reading frames and 11,097 SNPs were identified. Of those SNPs, 2,138 (19.3%) resulted in predicted amino acid substitutions.

To assess the reliability of these SNPs, we examined 15 contigs coding for genes related to metabolism and detoxification (Table 3). A total of 177 SNPs were found in 18,156 bp coding sequences examined. Among them, 65 were transition substitutions, resulting in 27 non-synonymous substitutions, whereas 112 SNPs were transversion substitutions, resulting in 31 non-synonymous substitutions. Overall, transitions resulted in more frequent non-synonymous substitutions (41.5%) than did transversions (27.7%). The average number of SNPs per kb ranged from 2.9 (carboxylesterase) to 33.8 (GSTE6).

**Table 3:** **Nucleotide polymorphism of genes related to metabolism, detoxification and immunity in** ***Anopheles sinensis***

Accession no.	Gene	Open reading frame	Chromosome*	Transition	Transversion	Total	Coverage	Average no. SNP per kb
GAFE01006078	CYP6AG2	148–1575	2R	2 (0)	12 (4)	14	11–29	9.8
GAFE01013675	CYP6ZX	193–1587	3R	8 (3)	11 (3)	19	10–25	13.6
GAFE01007218	CYP6Y1	330–1460	3R	2 (0)	4 (0)	6	11–20	5.3
GAFE01004582	GSTT1	63–749	X	0	3 (1)	3	18–34	4.4
GAFE01006360	GSTD5	94–735	2R	0	2 (0)	2	10–10	3.1
GAFE01012264	GSTE6	15–488	3R	10 (6)	6 (2)	16	10–17	33.8
GAFE01006339	Carboxylesterase	515–1204	2R	1 (0)	1 (0)	2	10–10	2.9
GAFE01005990	NYD-GBE	688–2178	3 L	1 (1)	7 (0)	8	12–26	5.4
GAFE01001512	Euk_Ferritin	390–1085	2R	5 (0)	6 (0)	11	25–110	15.8
GAFE01000429	LR1M1	46–1548	2 L	4 (3)	10 (5)	14	16–115	9.3
GAFE01003583	Serpin	116–1564	2R	8 (3)	15 (3)	23	10–35	15.9
GAFE01007678	GlgB	96–2156	3 L	12 (8)	7 (2)	19	11–278	9.2
GAFE01008565	Serine	481–2070	3R	1 (0)	4 (2)	5	11–14	3.1
GAFE01018362	Peroxinectin	364–2040	2R	9 (2)	8 (5)	17	10–24	10.1
GAFE01021559	Tubulin	263–1504	2 L	2 (1)	16 (4)	18	10–15	14.5

The number in parentheses indicates non-synonymous single nucleotide polymorphisms (SNPs).

*Chromosome assignment was based on synteny to *Anopheles gambiae*.

## Discussion

*Anopheles sinensis* is the most important malaria vector in China and other Southeast Asian countries due to its overwhelming dominance in abundance [1, 13, 57–59]. High levels of insecticide resistances and mechanisms to insecticide have been reported in *An. sinensis*[13, 14, 16, 18, 19]. The vector capacity and susceptibility of *An. sinensis* have been examined [3, 58, 60]. Recently, a preliminary genome sequence of *An. sinensis* was published [61]. However, the lack of transcriptome data has hindered research for molecular mechanisms of malaria transmission and new vector control methods. This study generated an EST database for *An. sinensis* and determined the transcripts associated with deltamethrin resistance. Our *de novo* assembly generated 33,411 contigs with average length of 493 bp. A total of 8,057 ESTs were generated with GO and KEGG annotation. Further, we identified more than 2,400 microsatellite markers. Therefore, this study has enhanced knowledge on the *An. sinensis* genome and transcriptome and has provided new tools for future genetic research

Although *de novo* assembly of 454 reads is more straight forward than Illumina reads, it may not be exhaustive. Published studies on mosquito species that used 454 sequencing include *An. funestus*, *Ae. aegypti* and *Ae. albopictus*[24, 62, 63]. The 454 sequencing in *An. funestus* generated 375,619 reads, and *De novo* assembly generated 18,103 contigs with average length of 253 bp [24]. In *Ae. aegypti*, 454 pyrosequencing resulted in 0.2 - 0.3 million useable reads and *De novo* assembly generated approximately 15,000 contigs [62]. With >1.1 million quality reads obtained, Poelchau et al. reported 69,474 contigs assembled in *Ae. albopictus* mosquitoes [63]. The present study obtained about 400,000 high-quality reads, and *De novo* assembly generated more than 33,000 contigs with a much higher average length, suggesting that our assembly for *An. sinensis* based 454 sequencing was reasonably good in comparison to the previously published transcript assembly.

RNA-seq allowed a holistic view of the transcriptome and provided absolute rather than relative gene expression measurements. Our RNA-seq analysis identified more than 2,100 transcripts that may be associated with deltamethrin resistance. Among them, 1,079 transcripts were up-regulated and 1,052 down-regulated. In *An. gambiae*, RNA-seq identified a total of 1,093 transcripts with significantly differential expression between deltamethrin resistant and susceptible mosquitoes from Kenya [41]. These transcripts were distributed over the entire genome, with some transcripts mapped within the previously identified quantitative trait loci (QTLs) linked to permethrin resistance, such as *rtp1* and *rtp2*[41]. The present study with *An. sinensis* detected twice the number of differentially expressed transcripts as in *An. gambiae*. The higher number of differentially expressed transcripts identified in *An. sinensis* may be partially due to the difference in deltamethrin resistance. The study populations used in the present study were extremely resistant, as evidenced by <10% morality in the standard WHO tube test for insecticide susceptibility, in contrast to >66% morality in *An. gambiae*. Further, metabolic detoxification enzymes were found to be very important in *An. sinensis*[16]. The present study confirmed that transcripts with the largest increase in expression were in metabolic pathways, particularly those coding for glutathione S-transferase, P450 monooxygenase, and cytochrome C oxidase detoxification enzymes. This is consistent with previous findings on higher P450 monooxygenase activities in pyrethroid resistant *An. gambiae*[24] and the association between the overexpression of cytochrome C oxidase subunit I gene and pyrethroid resistance in German cockroaches, *Blattella germanica*[64].

Our transcriptome analysis provided insights on the role of specific metabolic detoxification genes in resistance. For example, contig 6816 was annotated to be CYP6Z2 (access no GAFE01005115), and showed 89% similarity in amino acid sequence to AGAP008218 of *An. gambiae*, which was known to be one of the important detoxification genes responsible for pyrethroid resistance in *An. gambiae*[65, 66] and *An. arabiensis*[67, 68]. The present study found that this gene was strongly up-regulated in resistant mosquitoes and it showed the highest differentiation in expression between resistant and susceptible mosquitoes. The other cytochrome P450 genes that were strongly up-regulated in the resistant *An. sinensis* mosquitoes were CYP4 genes, including CYP4G16, CYP4G17 and CYP4H15. This is consistent with previous studies in house flies and other mosquito species that showed P450 genes were up-regulated in resistant individuals [69–72]. Similarly, we also found two GST genes (GSTU2 and GSTO1), 3 cytochrome C oxidase genes (COX2, COX5A and COX10), and 6 NADH dehydrogenase genes were significantly up-regulated. It is of interest to note that arrestin gene (ARR2, access no: GAFE01006709) and thioredoxin peroxidase gene (TPX2, access no: GAFE01001198) were found strongly up-regulated in the resistant mosquitoes, consistent with published reports in *Cx. pipiens pallens*[73], *An. arabiensis* and *An. gambiae*[66, 68, 74].

On the other hand, our study also identified some CYP6 genes were down-regulated in the resistant mosquitoes, including CYP6AG2, CYP6M3, CYP6P5 and CYPY1. Other cytochrome C oxidase genes (COX7C and COX1) were also down-regulated. Similar phenomenon was reported in *Cx. quinquefasciatus* mosquitoes [75]. The mechanisms for down-regulation of some P450 genes and their relevance to insecticide resistance are unclear. It has been suggested that reduction in the expression of some metabolic detoxification genes may result from responses to various endogenous and exogenous compounds, or to pathophysiological signals [75–78].

The present study identified cuticular proteins differentially expressed between deltamethrin resistant and susceptible *An. sinensis* mosquitoes. The cuticular proteins may play a role in insecticide resistance by limiting the penetration of insecticides into the mosquito cuticle. This finding is not unique to *An. sinensis*; similar observations were reported in *An. funestus*. It is possible that thicker mosquito cuticles slow insecticide absorption and consequently increase the efficiency of metabolic detoxification [21]. It is important to note that while a transcript may have a large difference in expression between resistant and susceptible mosquitoes, this does not necessarily imply causality in the gene. Therefore, it is important to identify the key candidate genes, and the function of the candidate transcripts on pyrethroid resistance should be confirmed independently.

Molecular markers play an important role in genetic mapping, population genetics and genomics. In this study, we performed a genome-wide scan on the *An. sinensis* transcripts for microsatellites markers. A search for di- to hexa-nucleotide repeats yielded a total of 2,408 potential microsatellite markers. As expected, di-nucleotide repeats were the most frequent microsatellite motifs, followed by trinucleotide repeats. AC/GT (29.7%) and AGC (8.3%) were the most frequent motifs among the di-nucleotide and tri-nucleotide microsatellites respectively. We identified a total of 15,496 potential SNPs in the coding sequencing, or approximately 6 SNPs per 1000 bp. Such a SNP density was comparable to *Aedes aegypti*[79] and *An. gambiae*[80, 81] with 5–9 SNPs per 1000 bp, but was substantially lower than *An. funestus* that had an average of 14 SNPs per 1000 bp [24]. The rate of transition (60%) and transversion (40%) substitutions was similar to *An. funestus*[24, 82], Copepod [83] and fish [84]. The frequency of non-synonymous substitution SNPs was approximately 20%, similar to human (22%) [85]. These SNPs and microsatellite markers provide an extensive set of genetic markers for *An. sinensis,* and will facilitate future population genetic studies of this important malaria vectors in Southeast Asia.

## Conclusion

This study has generated an EST database for *An. sinensis* mosquitoes and enriched the genetic information for this important malaria vector species. RNA-seq analysis identified differentially expressed transcripts, particularly the transcripts coding for metabolic detoxification enzymes and cuticular proteins. The microsatellite and SNP markers identified here provide new tools for future population and evolutionary genetics research.

## Electronic supplementary material

Additional file 1: **Mortality rate of deltamethrin resistance bioassay and** ***kdr*** **allele frequency in four** ***Anopheles sinensis*** **mosquito populations from Jiangsu Province, China.** (DOCX 18 KB)

Additional file 2: **The taxonomy distribution of the contigs matched to the nr databases by BLASTX.** (XLSX 21 KB)

Additional file 3: **Gene Ontology (GO) accession number assignment of the 8057 ESTs.** (XLSX 1 MB)

Additional file 4: **A list of significantly differentially expressed genes between field resistant and laboratory susceptible adult mosquitoes, and between field resistant and field susceptible adult mosquitoes.** Relative expression levels and fold changes were log2 (RPKM + 0.0001) transformed. Negative value indicates down-regulation and positive value indicates up-regulation in the resistant population. Threshold for significant expression: absolute value of Log2 (ratio) > 1, P < 0.01 and FDR corrected value (q) < 0.01. (XLSX 341 KB)

Additional file 5: **KO annotations of the genes with the largest difference in expression between deltamethrin resistant and susceptible** ***Anopheles sinensis*** **mosquitoes.** Transcripts 1–10 are the genes with increased expression in the resistant mosquitoes, and transcripts 11–20 are the genes with reduced expression in the resistant mosquitoes. (PPTX 93 KB)

Additional file 6: A list of gene name, function and primers used in qRT-PCR amplification for RNA-seq validation. (DOCX 21 KB)

Additional file 7: **Correlation of expression value measured by RNA-seq and qRT-PCR.** The fold change in RNA-seq was measured by the log2 of RPKM (reads per kilobase per million mapped reads). The fold change in qRT-PCR was measured by ΔC_T_ value. (PPTX 47 KB)
