# Supplementary material for: Transcriptome profiling of pyrethroid resistant and susceptible mosquitoes in the malaria vector, Anopheles sinensis
Source: BMC Genomics. 2014 Jun 9;15(1):448. doi: 10.1186/1471-2164-15-448 (PMC4070547; doi:10.1186/1471-2164-15-448)
Supplement: Supplementary file 1 — Additional file 1: Mortality rate of deltamethrin resistance bioassay and kdr allele frequency in four Anopheles sinensis mosquito populations from Jiangsu Province, China. (DOCX 18 KB) [file 12864_2013_6125_MOESM1_ESM.docx]

### Additional_file_1: Moratlity rate of deltamethrin resistance bioassay and *kdr* allele frequency in four *Anopheles sinensis* mosquito populations from Jiangsu Province, China.

| Population | n | Mortality ± standard error (%)* | Insecticide status** | *kdr* allele frequency (%)  (L1014F and L1014C) |
| --- | --- | --- | --- | --- |
| Luhe, Nanjing | 136 | 4.4 ± 3.5 | Resistant | 88.2 |
| Suining, Xuzhou | 120 | 7.5 ± 4.7 | Resistant | 98.3 |
| Yixing, Wuxi | 135 | 3.7 ± 3.2 | Resistant | 97.8 |
| Wujin, Changzhou | 123 | 8.2 ± 4.8 | Resistant | 100 |
| Laboratory susceptible strain | 100 | 100 |  | 0 |
| Control*** | 40 | 0 |  |  |

* Mortality refers to the percentage of mosquitoes that died 24 hrs after recovery from a 60 min exposure to the insecticides.

** Resistance status is based on WHO 2013 criteria: ‘‘Resistant’’ (mortality less than 90%), ‘‘Probable Resistant’’ (mortality rate ranged from 90–97%), and ‘‘Susceptible’’

(mortality more than 98%).

*** Twenty mosquitoes of lab strain were exposed to filter papers without insecticide for 60 min, and mortality was recorded after the 24 hrs recovery period; two replicates.
