# Supplementary material for: Transcriptome profiling of pyrethroid resistant and susceptible mosquitoes in the malaria vector, Anopheles sinensis
Source: BMC Genomics. 2014 Jun 9;15(1):448. doi: 10.1186/1471-2164-15-448 (PMC4070547; doi:10.1186/1471-2164-15-448)
Supplement: Supplementary file 5 — Additional file 5: KO annotations of the genes with the largest difference in expression between deltamethrin resistant and susceptible Anopheles sinensis mosquitoes. Transcripts 1–10 are the genes with increased expression in the resistant mosquitoes, and transcripts 11–20 are the genes with reduced expression in the resistant mosquitoes. (PPTX 93 KB) [file 12864_2013_6125_MOESM5_ESM.pptx]

## Slide 1
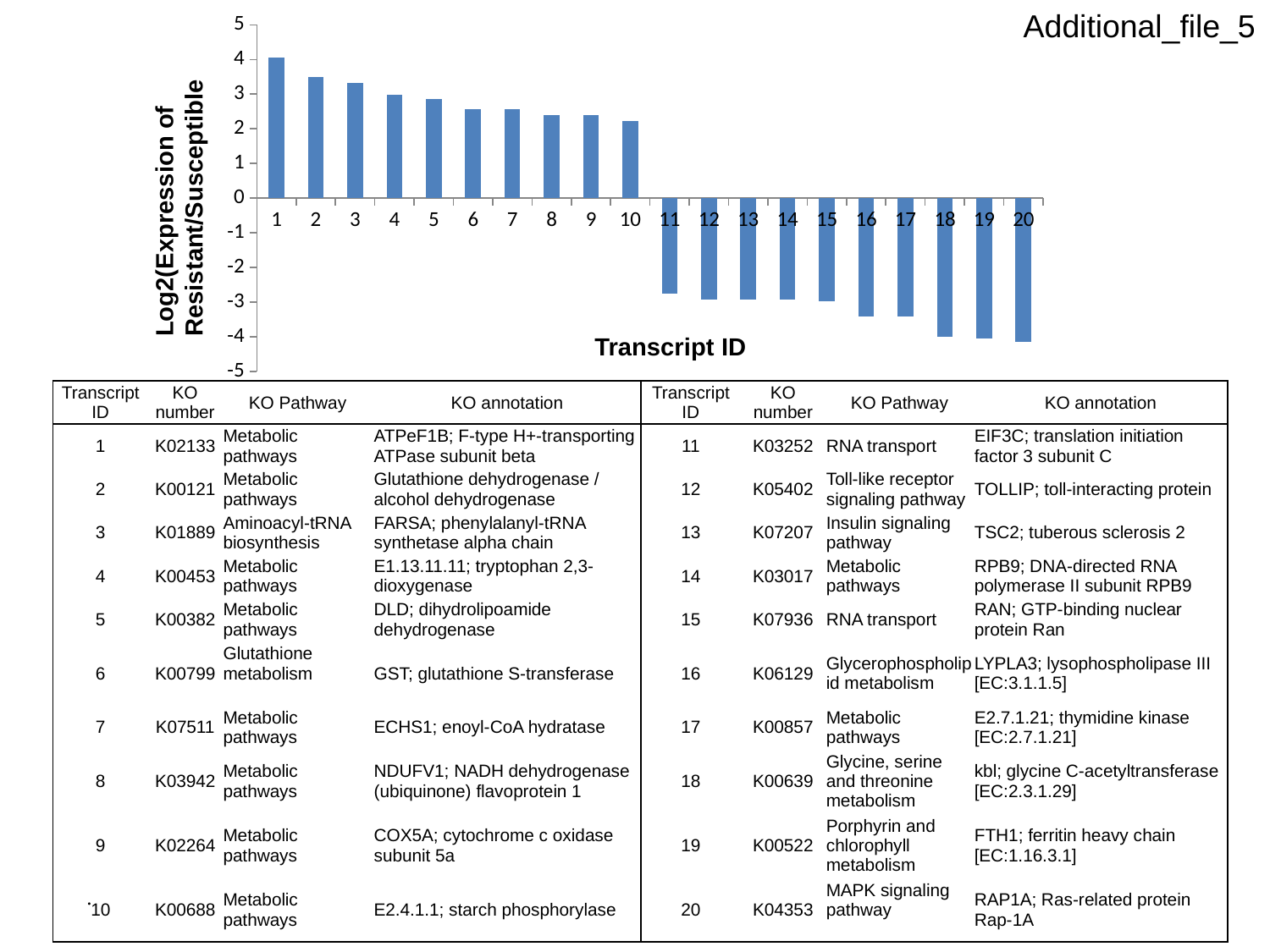

Additional_file_5
### Chart
| Category | |
|---|---|Log2(Expression of Resistant/Susceptible
Transcript ID
| Transcript ID | KO number | KO Pathway | KO annotation | Transcript ID | KO number | KO Pathway | KO annotation |
| --- | --- | --- | --- | --- | --- | --- | --- |
| 1 | K02133 | Metabolic pathways | ATPeF1B; F-type H+-transporting ATPase subunit beta | 11 | K03252 | RNA transport | EIF3C; translation initiation factor 3 subunit C |
| 2 | K00121 | Metabolic pathways | Glutathione dehydrogenase / alcohol dehydrogenase | 12 | K05402 | Toll-like receptor signaling pathway | TOLLIP; toll-interacting protein |
| 3 | K01889 | Aminoacyl-tRNA biosynthesis | FARSA; phenylalanyl-tRNA synthetase alpha chain | 13 | K07207 | Insulin signaling pathway | TSC2; tuberous sclerosis 2 |
| 4 | K00453 | Metabolic pathways | E1.13.11.11; tryptophan 2,3-dioxygenase | 14 | K03017 | Metabolic pathways | RPB9; DNA-directed RNA polymerase II subunit RPB9 |
| 5 | K00382 | Metabolic pathways | DLD; dihydrolipoamide dehydrogenase | 15 | K07936 | RNA transport | RAN; GTP-binding nuclear protein Ran |
| 6 | K00799 | Glutathione metabolism | GST; glutathione S-transferase | 16 | K06129 | Glycerophospholipid metabolism | LYPLA3; lysophospholipase III [EC:3.1.1.5] |
| 7 | K07511 | Metabolic pathways | ECHS1; enoyl-CoA hydratase | 17 | K00857 | Metabolic pathways | E2.7.1.21; thymidine kinase [EC:2.7.1.21] |
| 8 | K03942 | Metabolic pathways | NDUFV1; NADH dehydrogenase (ubiquinone) flavoprotein 1 | 18 | K00639 | Glycine, serine and threonine metabolism | kbl; glycine C-acetyltransferase [EC:2.3.1.29] |
| 9 | K02264 | Metabolic pathways | COX5A; cytochrome c oxidase subunit 5a | 19 | K00522 | Porphyrin and chlorophyll metabolism | FTH1; ferritin heavy chain [EC:1.16.3.1] |
| 10 | K00688 | Metabolic pathways | E2.4.1.1; starch phosphorylase | 20 | K04353 | MAPK signaling pathway | RAP1A; Ras-related protein Rap-1A |
.
