# Supplementary material for: Transcriptome profiling of pyrethroid resistant and susceptible mosquitoes in the malaria vector, Anopheles sinensis
Source: BMC Genomics. 2014 Jun 9;15(1):448. doi: 10.1186/1471-2164-15-448 (PMC4070547; doi:10.1186/1471-2164-15-448)
Supplement: Supplementary file 6 — Additional file 6: A list of gene name, function and primers used in qRT-PCR amplification for RNA-seq validation. (DOCX 21 KB) [file 12864_2013_6125_MOESM6_ESM.docx]

### Additional_file_6: A list of gene name, function and primers used in qRT-PCR amplification for RNA-seq validation.

| **Access** | **Genes** | **Function** | **Forward Primers** | **Reverse Primers** | **Product size (bp)** |
| --- | --- | --- | --- | --- | --- |
| GAFE01005115 | CYP6Z2 | Cytochrome P450 protein | ATCGAGGCGTCTACTGCAAC | AATTGGCCCGAGGTGAAG | 121 |
| GAFE01015622 | CYP4H15 | Cytochrome P450 protein | TGGGACACTGCAAGAGCTAA | CCATATCTCCAGCCGATCAC | 103 |
| GAFE01015818 | ACE1 | Acetylcholinesterases | GCGACGAGATCAACTACGTG | CTTGGCAAAGTTGGACCAGT | 113 |
| GAFE01007916 | CPR15 | Cuticle protein | AGGGCAGCTACTCGTACACC | ATCTGCTCGAGCGTCTTCA | 145 |
| GAFE01023437 | TOLL9 | Toll protein | CGATGTTGCAATAGCTGACG | CCTGACGTTCCTCGATCTGT | 104 |
| GAFE01003138 | CLIPA6 | Clip-domain serine protease | TCCAGCTGAACGAGGGTATC | TTCCGTACACGTCCTTACCC | 102 |
| GAFE01007287 | GSTU2 | G[lutathione S-transferase](http://www.ncbi.nlm.nih.gov/nuccore/NM_001046665.1) | TCTATCTCGGCGAGTCACG | GATCATGGCTTTCTCCTTCG | 101 |
| GAFE01000064 | 18S | Ribosomal protein | TCGAAGGCGATTAGATACCG | CCGGAAGCTACTGAGAGCAC | 95 |
