# Supplementary figures and images for: Transcriptome profiling of pyrethroid resistant and susceptible mosquitoes in the malaria vector, Anopheles sinensis
Source: BMC Genomics. 2014 Jun 9;15(1):448. doi: 10.1186/1471-2164-15-448 (PMC4070547; doi:10.1186/1471-2164-15-448)

## Slide 1
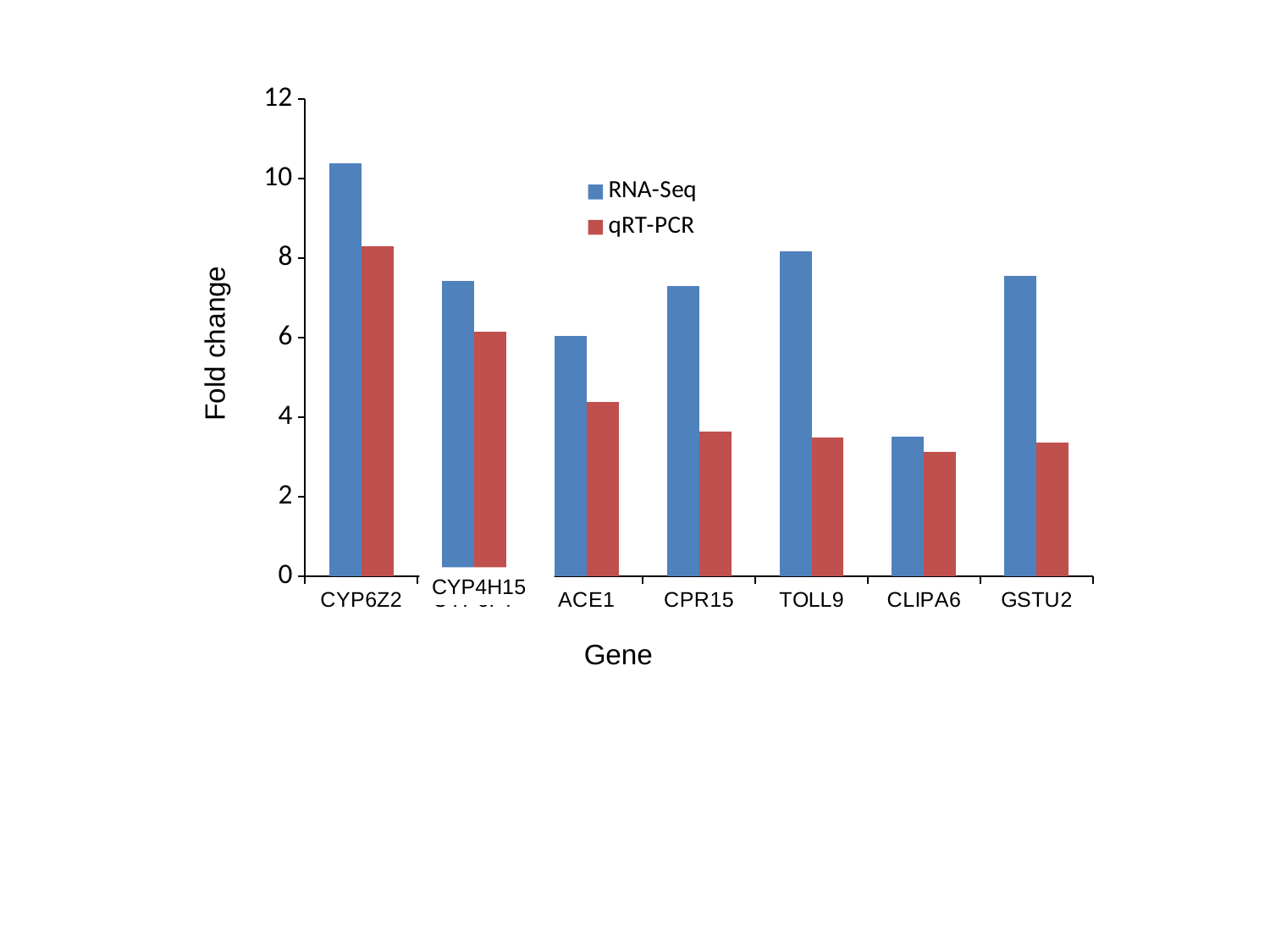

### Chart
| Category | RNA-Seq | qRT-PCR |
|---|---|---|
| CYP6Z2 | 10.382472724779387 | 8.293194999999999 |
| CYP6P7 | 7.430000854687776 | 6.143307500000001 |
| ACE1 | 6.036701130285361 | 4.3830275 |
| CPR15 | 7.298154237247633 | 3.6349324999999997 |
| TOLL9 | 8.16581649286144 | 3.48685 |
| CLIPA6 | 3.4939444797772987 | 3.114 |
| GSTU2 | 7.544902629268257 | 3.364175 |Fold change
CYP4H15
Gene

Supplement: Supplementary file 7 — Additional file 7: Correlation of expression value measured by RNA-seq and qRT-PCR. The fold change in RNA-seq was measured by the log2 of RPKM (reads per kilobase per million mapped reads). The fold change in qRT-PCR was measured by ΔCT value. (PPTX 47 KB) [file 12864_2013_6125_MOESM7_ESM.pptx]
